# Functionalizing cell-mimetic giant vesicles with encapsulated bacterial biosensors

**DOI:** 10.1098/rsfs.2018.0024

**Published:** 2018-08-17

**Authors:** Tatiana Trantidou, Linda Dekker, Karen Polizzi, Oscar Ces, Yuval Elani

**Affiliations:** 1 Department of Chemistry, Imperial College London, London SW7 2AZ, UK; 2 Department of Life Sciences and Centre for Synthetic Biology and Innovation, Imperial College London, London SW7 2AZ, UK; 3 Institute of Chemical Biology, Imperial College London, London SW7 2AZ, UK; 4 fabriCELL, Imperial College London, London SW7 2AZ, UK

**Keywords:** giant lipid vesicles, synthetic biology, biosensing, microfluidics, artificial cells, cellular bionics

## Abstract

The design of vesicle microsystems as artificial cells (bottom-up synthetic biology) has traditionally relied on the incorporation of molecular components to impart functionality. These cell mimics have reduced capabilities compared with their engineered biological counterparts (top-down synthetic biology), as they lack the powerful metabolic and regulatory pathways associated with living systems. There is increasing scope for using whole intact cellular components as functional modules *within* artificial cells, as a route to increase the capabilities of artificial cells. In this feasibility study, we design and embed genetically engineered microbes (*Escherichia coli*) in a vesicle-based cell mimic and use them as biosensing modules for real-time monitoring of lactate in the external environment. Using this conceptual framework, the functionality of other microbial devices can be conferred into vesicle microsystems in the future, bridging the gap between bottom-up and top-down synthetic biology.

## 1. Introduction

In recent years, there has been an upsurge in interest surrounding the design and construction of functional vesicle-based systems. The application drivers for this area of research are numerous: from the use of vesicles for drug delivery [1–3], sensing agents [1,4] and microreactors [5–7] to their employment as chassis for artificial cells [8,9] and tissue mimics [10].

Vesicles are composed of a structural fabric of either phospholipids or fatty acids [11]. However, the components responsible for their functionality are more diverse. These include: ligands that decorate the membrane (e.g. DNA sticky ends, antibodies, tethered small molecules, nanoparticles, polymerizable amphiphiles) [7,12–15]; membrane-spanning structures (e.g. natural and engineered protein pores, pumps and channels, DNA origami) [16–18]; and biologically derived material encapsulated in the vesicle lumen (e.g. enzymes, cytoskeleton components, polymerases and coupled transcription/translation machinery) [6,19–22]. This varied repertoire of building blocks has allowed investigators to engineer vesicles capable of mimicking an assortment of cellular behaviours [23], and of successfully interfacing cell and vesicle communities together [24].

Recently, a new front in the construction of vesicle-based microsystems has opened up with the emergence of technologies allowing the encapsulation of whole cells within vesicles [25,26] using emulsion transfer vesicle generation methods [27]. Our previous work of encapsulating *non-engineered* cells in this context [25,26] paves the way for the rich and mature world of synthetic biology and genetic engineering to be tapped for the assembly of vesicles with enhanced functionality, using encapsulated cells as embedded functional modules. Encapsulated cells, for example, could be used in the design of compartmentalized systems capable of exhibiting sensor and bioreactor modalities, of harvesting energy and of dynamically responding to environmental stimuli.

Herein, we demonstrate this potential by designing genetically engineered microbes and using them as biosensing modules that are encapsulated within a vesicle chassis. Increasingly, synthetic biologists are exploiting the diverse array of sensors and regulators in nature that aid in survival to create devices that fill the growing need for low-cost diagnostic tools in medical [28], environmental [29] and manufacturing [30] applications. Demonstrating the validity of this conceptual framework shows that the functionality of other microbial devices can be conferred into vesicle microsystems in the future.

Encapsulating engineered organisms in vesicles allows them to be shielded from their external environment, which can be useful in several applications. For example, when using bacterial biosensors in co-cultures, encapsulation enables the sensor cells and the main culture cells to be physically separated, preventing unwanted interference and allowing the two species to exist in distinct optimized conditions. This is also true of potential medicinal applications; for example, in cell-based therapies, where encapsulation could shield the cell from the host's immune response. The potential for facile precision engineering of an external membrane using biological and synthetic components is also attractive for these applications; for example, the incorporation of molecular recognition modules, stimuli-responsive membranes, protein channels and functionalized nanoparticles. In short, hybrid systems such as these allow the advantages associated with both bottom-up and top-down synthetic biology to be combined.

There have been efforts in encapsulating engineered cells in droplets [31], gels [32], hydrogel microcapsules [33] and liquid core/shell structures [34], but not within cell-mimetic vesicles, which, owing to their biocompatibility, similarity to cell membranes and the potential for bilayer functionalization, are the most commonly used artificial cell chassis. We demonstrate this feature by introducing transmembrane pores into the vesicle membrane to facilitate the influx of analyte to the vesicle interior.

As a proof of concept, we created a whole-cell *Escherichia coli* lactate biosensor based on the transcriptional elements of the lldPRD operon that produces green fluorescent protein (GFP) in response to lactate. Lactate was chosen as an exemplar system because it is of interest in a variety of different industrial and biotechnological applications. For example, in bioprocessing, lactate is the primary waste metabolite and can negatively affect cell growth and productivity; therefore, it needs to be monitored to help control the culture trajectory and ensure product quality [30]. We show that the encapsulated biosensors can sense the external lactate concentration by incorporating membrane-spanning α-haemolysin (α-HL) pores embedded in the vesicle membrane, allowing diffusion of the analyte to the vesicle interior. Once the lactate diffuses across the cell membrane, the LldR regulatory protein binds to it and induces the lldPRD promoter, which then drives the expression of GFP leading to measurable fluorescence. This feasibility study demonstrates the potential of *cellular bionics*, where ‘living’ modules are fused with artificial cell microsystems for enhanced functionality.

## 2. Material and methods

### 2.1. Biosensor construction

The lactate biosensor was modified from Goers *et al*. [35], where the lldPRD promoter was modified to include the J23117 constitutive promoter within the O1 and O2 sites (electronic supplementary material, table S1). Strains were maintained on Luria–Bertani (LB) agar containing 100 µg ml^−1^ ampicillin and grown aerobically for 16 h at 37°C.

### 2.2. Biosensor characterization in liquid medium

**Figure 1.**
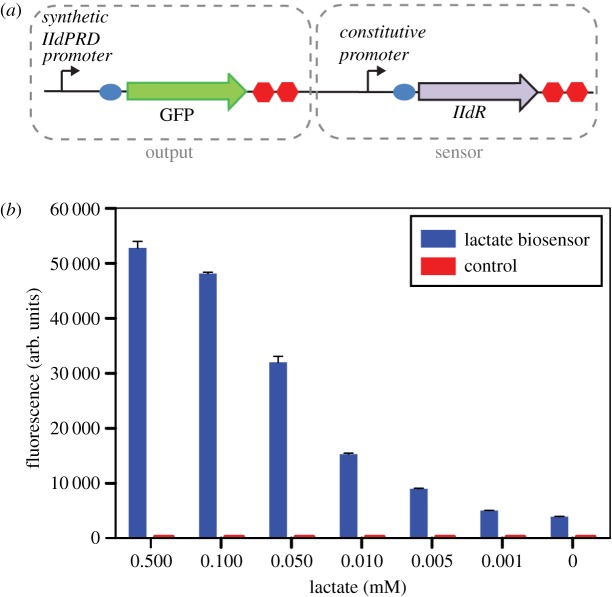
Lactate biosensor used in this study. (*a*) Diagram of the lactate biosensor in *E. coli*. (*b*) Characterization of lactate biosensor in bulk (non-encapsulated form) using a flow cytometer. The whole-cell *E. coli* biosensor and control cells were spiked with different concentrations of l-lactate.

Single colonies of *E. coli* containing the biosensor plasmid or a control plasmid (lacking the output module of the biosensor (figure 1*a*)) were used to inoculate 2 ml of LB medium containing 100 µg ml^−1^ ampicillin and grown at 37°C with shaking at 250 rpm for approximately 6 h. The starter culture was diluted 1 : 100 into 5 ml of M9 minimal medium (1× M9 salts, 2 mM MgSO_4_, 0.1 mM CaCl_2_, 0.34 g l^−1^ thiamine hydrochloride, 1 g l^−1^ NH_4_Cl and 0.4% glycerol) containing 100 µg ml^−1^ ampicillin and grown overnight at 37°C with shaking. The optical density of the overnight culture was adjusted to an OD_600 nm_ of 1.0 in M9 minimal medium. In a 96-well plate, 25 µl of adjusted culture was added to a well containing 87.5 µl of M9 minimal medium containing 100 µg ml^−1^ ampicillin. The plate was covered with a Breathe-Easy^®^ seal (Sigma Aldrich) and incubated at 37°C with shaking for 2 h for cells to reach exponential growth phase. Lactate (12.5 µl) of varying concentrations dissolved in M9 minimal medium was added to each well. The plate was covered with a Breathe-Easy^®^ seal and incubated at 37°C with shaking for 2 h. Samples (20 µl) were taken from each well and mixed with 180 µl of filtered sterile water. Fluorescence in each well was measured using an Attune^®^ Acoustic Focusing Cytometer (ThermoFisher; excitation 485 nm, emission 528 nm). Data were analysed using FlowJo software (FlowJo LLC, Ashland, OR).

### 2.3. Biosensor preparation for encapsulation

The biosensor was grown under the same conditions as for the characterization studies (above). Following incubation for 2 h at 37°C with shaking, 2 ml of *E. coli* cells in exponential phase containing the biosensor were concentrated by centrifugation and resuspended in 600 µl of medium, yielding a concentration of approximately 5 × 10^8^ cells ml^−1^.

### 2.4. Vesicle formation and biosensor assay

All lipids were purchased from Avanti Polar Lipids (USA) and were used without further purification. Lactate oxidase, horseradish peroxidase (HRP) and Amplex Red were purchased from Life Technologies (USA). Lactate, α-HL and all other chemicals were purchased from Sigma Aldrich (UK) unless otherwise stated. Giant unilamellar vesicles (GUVs) were generated using the phase transfer of individual droplets across a water–oil interface by modifying a procedure described elsewhere [36]. Sodium l-lactate, 99% purity, pH 7.4 in M9 minimal medium, was used.

A POPC (1-palmitoyl-2-oleoyl-*sn*-glycero-3-phosphocholine) lipid film was first prepared by dissolving 2 mg of lipid in chloroform and removing the chloroform under a stream of nitrogen and placing in a lyophilizer for 1 h. This was dissolved in mineral oil (2 mg ml^−1^) by placing the vial in a 60°C oven for 1 h.

A 0.2 ml aliquot of POPC in mineral oil solution was then deposited above 0.2 ml of 500 mM glucose in M9 minimal medium in an Eppendorf tube to form a column. The ‘internal’ GUV solution was then prepared, which was composed of sucrose (450 mM), lactate (0–50 mM) and engineered *E. coli* (1 : 9 dilution of 5 × 10^8^ cells ml^−1^); 20 µl of this solution was added to 200 µl of lipid in oil and an emulsion was made by vortexing for 30 s. This emulsion was then layered above the water–oil column, and the tube centrifuged to transform the emulsion drops into lipid vesicles. The non-encapsulated material was removed from the surrounding solution by pelleting the GUVs three times (500*g*; 5 min), removing the supernatant and re-suspending in medium. This process was repeated three times to remove the majority of non-encapsulated bacteria. α-HL was added to the GUVs at a 1 : 9 ratio to give a final α-HL concentration of 0.05 mg ml^−1^. Lactate was then added to the external solution at a 1 : 9 ratio to give final concentrations ranging from 0 to 50 mM. The effectiveness of the biosensor/vesicle hybrid was characterized using a fluorimeter to monitor GFP production (excitation 485/20 nm; emission 528/20 nm; Synergy HT; BioTek Instruments, Inc., Winooski, VT, USA) and five replicates were measured for each condition.

### 2.5. Lactate enzymatic assay

For the lactate enzymatic assay, GUVs were formed as before, but with different inner and outer solutions. All aqueous solutions were prepared in phosphate-buffered saline. The internal vesicle solution consisted of 1 U ml^−1^ lactate oxidase and 450 mM sucrose. The external solution was composed of 500 mM arabinose solution to form a column. This was then centrifuged at 9000*g* for 30 min, allowing the water droplets in the emulsions to transfer from the oil to water phase, thus forming vesicles that aggregated into a pellet. The supernatant was removed, the pellet resuspended in 500 µl arabinose, and three more centrifugation rounds applied to remove any non-encapsulated enzyme. The pellet was resuspended in 450 mM sucrose solution. α-HL was then added at a 1 : 9 ratio to give a final α-HL concentration of 0.05 mg ml^−1^; 80 µl of this GUV solution was placed in a single well of a 96-well plate, followed by addition of 5 µl HRP (10 U ml^−1^), 5 µl Amplex Red (10 mM) and 10 µl lactate to give final lactate concentrations ranging from 0 to 1000 µM. Fluorescence was monitored using a fluorimeter (excitation 485/20 nm; emission 528/20 nm; Synergy HT) and five repeats were obtained for each condition.

### 2.6. Microfluidic encapsulation of bacteria

Polydimethylsiloxane (PDMS) microfluidic devices consisting of a single flow-focusing junction were fabricated via soft lithography as described elsewhere [37]. Briefly, negative masters were produced using SU-8 (A-Gas Electronic Materials, UK) photoresist and standard lithography. After development using EC solvent (A-Gas Electronic Materials, UK), the negative masters were exposed to trichloro(1H,1H,2H,2H-perfluorooctyl)silane vapour to suppress permanent adhesion to moulded PDMS. PDMS prepolymer and curing agent were then thoroughly mixed in a 10 : 1 ratio, and the mixture was poured onto the master wafer. The mixture was cured at 65°C overnight, then peeled off the master and bonded on PDMS-coated microscope glass slides (75 × 25 mm; VWR, UK) via plasma oxidation (100 W, 1 min, 20 sccm O_2_ flow, 67 Pa pressure) using a Femto plasma cleaner (Diener Electronic, Germany).

The microfluidic device was set up with the two fluid inlets and one vesicle outlet. The internal aqueous phase consisted of bacteria diluted at a 1 : 9 ratio with lactate (0–50 mM) present in M9 medium solution containing 450 mM sucrose. The bacteria–lactate solutions were mixed immediately before being inserted into the microfluidic device. The external oil phase consisted of FluorinertR™ FC-70 oil with 0.5% Pico-Surf™ (5% in FC-40) in FC-70 (Dolomite Microfluidics, UK). The internal aqueous phase and oil phase were injected using 1 ml plastic syringes linked to 1.09 mm polytetrafluoroethylene tubing (Adtech Polymer Engineering Ltd, UK). Two syringe pumps (Chemyx Inc., UK) were necessary to pump the reagents into the microfluidic system at controlled flow rates (2 and 3 µl min^−1^ for the water and oil phase, respectively). Droplets were generated at the flow-focusing junction and carried through the meander channel to the large reservoir. To examine fluorescence from the droplets, both flows were simultaneously stopped and the outlet was plugged to immobilize further movement of the droplets.

### 2.7. Fluorescence microscopy

Fluorescence images of encapsulated bacteria were viewed with an Olympus IX81 inverted microscope. Fluorescence experiments used an illuminating mercury arc lamp and were imaged with a fluorescein isothiocyanate filter, at an exposure time of 1000 ms. The lamp was turned off after each acquisition event to minimize photobleaching. Images were taken with a QICAM camera (QImaging).

## 3. Results and discussion

### 3.1. Lactate biosensor characterization

The lactate biosensor was constructed using a synthetic lldPRD promoter (figure 1*a*) that combines the natural lldPRD operator sites with a weak constitutive promoter. We first assessed its ability to detect lactate in samples spiked with different concentrations of l-lactate in M9 minimal medium containing glycerol as the carbon source. The lactate biosensor containing the synthetic lldPRD promoter exhibited higher fluorescence levels than a previous version containing the natural lldPRD promoter [35], which resulted in an increased limit of detection of lactate (electronic supplementary material, figure S1). The biosensor displayed a clear response to lactate with separation between different concentrations ranging from 0.5 to 0.005 mM (figure 1*b*). Control cells that lacked the output part of the biosensor did not fluoresce in the presence of lactate.

### 3.2. Vesicle engineering

**Figure 2.**
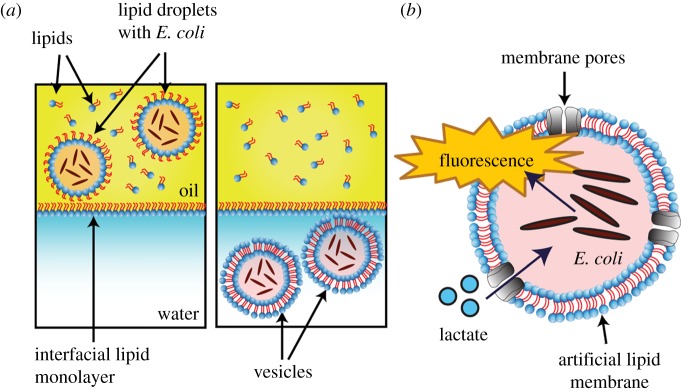
Hybrid lactate biosensor construction via the phase transfer of water-in-oil droplets. (*a*) Bacteria were encapsulated in lipid-coated water-in-oil droplets loaded with a mixture of sucrose and cell medium, and were expelled above a water–oil column stabilized with a lipid monolayer. The bacteria-loaded droplets sank through the column due to their higher density and picked up another lipid monolayer, forming lipid vesicles. (*b*) Schematic of the vesicle system that was generated for the hybrid lactate biosensor. Lactate permeates the vesicle via the α-HL pores and initiates a fluorescence response (GFP) from the encapsulated *E. coli*.

To encapsulate bacteria within giant vesicles, we took advantage of emulsion phase transfer technologies [27,36]. Lipid-stabilized water-in-oil emulsion droplets containing bacteria were transferred by gravity from an oil to an aqueous solution of a water–oil column, with a lipid monolayer present at the interface of the two phases. This process, driven by the higher density of the sucrose-containing droplets, led to the individual droplets being engulfed by a bilayer, resulting in a vesicle (figure 2*a*). The number of bacteria encapsulated in each GUV was controlled by adjusting the bacteria concentration beforehand (5 × 10^8^ cells ml^−1^, yielding approx. 70 bacteria in a 25 nl GUV).

The system required the construction of GUVs that are permeable to lactate, which otherwise cannot cross the vesicle membrane due to its negative charge. To do this, we decorated the GUVs composed of POPC lipid with transmembrane α-HL protein pores. This allowed lactate to reach the vesicle lumen and activate the bacterial biosensor (figure 2*b*) [38]. Protein monomers spontaneously insert into bilayers and aggregate to form heptameric protein pores of 1.4 nm diameter. In this way, the bacteria remain spatially separated from their external environment, but still retain their ability to sense and respond to the presence of lactate.

To test successful influx of lactate, we used an enzymatic assay based on the oxidation of lactate using lactate oxidase [39]. This yields H_2_O_2_, which subsequently oxidizes the non-fluorescent Amplex Red to the fluorescent molecule resorufin in the presence of HRP. GUVs were formed with lactate oxidase in the interior, α-HL embedded in the membrane, and HRP and Amplex Red and varying concentrations of lactate in the exterior (electronic supplementary material, figure S2a). The presence of α-HL allowed lactate oxidase in the vesicle interior to be exposed to lactate, with the H_2_O_2_ product (membrane permeable) diffusing out of the GUV to initiate subsequent steps of the cascade.

An increase in fluorescence was observed within approximately 20 min, with the reaction completing after approximately 120 min. This shows the successful flow of lactate through the pore, allowing it to be oxidized by lactate oxidase which is trapped in the GUV. With no α-HL, minimal fluorescence increase was seen, demonstrating that lactate oxidase was not present in significant amounts in the GUV exterior (e.g. due to vesicle rupture) and confirming that lactate cannot cross the vesicle membrane without α-HL pores. Larger lactate concentration gave higher fluorescence signals, with a linear relationship being present from 0 to 100 µM (electronic supplementary material, figure S2b). Above this concentration, the signal began to plateau (electronic supplementary material, figure S2c).

### 3.3. Response of GUVs functionalized with *Escherichia coli* lactate biosensors

**Figure 3.**
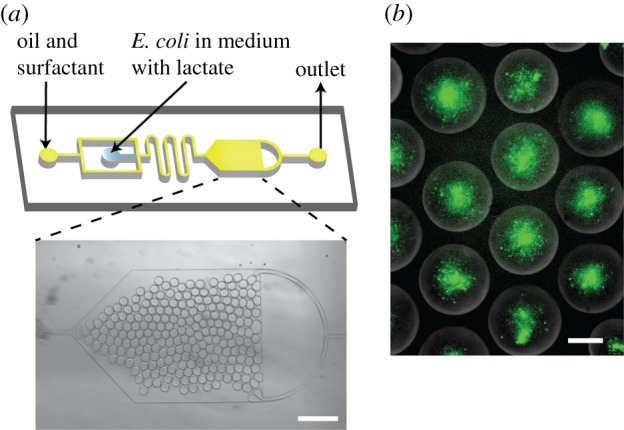
Microfluidic encapsulation of *E. coli* biosensor in droplets. (*a*) Microfluidic device design consisting of a flow-focusing junction and a large reservoir. Channel depth is 20 µm. Micrograph of the reservoir containing water-in-oil droplets. Scale bar, 400 µm. (*b*) Fluorescence images of bacteria encapsulated in water-in-oil droplets with 50 mM concentration after 300 min of incubation. Scale bars, 40 µm.

All biosensor experiments were conducted in M9 medium, and with 450 mM sucrose, as this was used to provide the density difference for vesicle generation via phase transfer. We first tested the system in monodisperse water-in-oil droplets on a microfluidic chip using bacteria that were premixed with varying concentrations of lactate. This was used to obtain the experimental conditions for subsequent experiments, and was particularly useful given that droplets are the precursors to the lipid vesicles themselves, and bacteria, therefore, exist in the same physiological environment. A PDMS microfluidic device with one flow-focusing junction was fabricated via standard soft lithography as described elsewhere [37]. The microfluidic device contained an observation chamber to accumulate droplets and facilitate the microscopy experiments (figure 3).

Bacteria were mixed with lactate (9 : 1) in M9 medium and were immediately encapsulated in droplets. Droplet sizes were monodisperse, with mean radii of 15 µm and the coefficient of variation less than 3%. Some droplets (less than 1%) were found to fuse with adjacent ones. Prototyping experiments revealed successful GFP production in the confined environment, with lactate concentrations ranging from 0.5 to 50 mM, at room temperature, over a period of 5 h.

**Figure 4.**
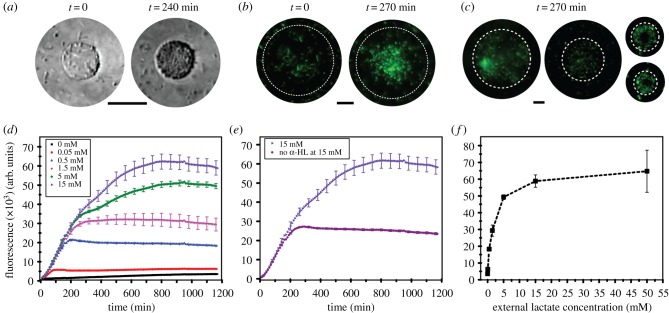
Full operational system showing the hybrid lactate biosensor. (*a*) Bright field images showing encapsulated microbes in vesicles over 240 min. Scale bar, 20 µm. (*b*) Fluorescence images of the hybrid lactate biosensor at 50 mM concentration of lactate at *t* = 0 and *t* = 270 min. Brightness and contrasts enhanced equally across the fluorescence images. Scale bar, 20 µm. (*c*) Fluorescence images of hybrid biosensors at 50 mM lactate concentration at *t* = 240 min. Scale bar, 20 µm. (*d*) Graph showing fluorescence intensity over time of the produced GFP at different lactate concentrations as lactate penetrates the vesicle system via the α-HL pores. The full two-step reaction cascade takes place. (*e*) Fluorescence signal at 15 mM lactate in the presence and absence of α-HL pores. (*f*) Characterization data of the hybrid biosensor at different concentrations of lactate. Error bars represent the standard deviation of five technical replicates.

Following these experiments, we encapsulated bacteria in GUVs with embedded α-HL pores in their lipid bilayers. The GUVs were subsequently inserted into external solutions of varying lactate concentrations: 0, 0.05, 0.5, 1.5, 5, 15 and 50 mM. Lactate diffused through the α-HL pores and initiated a fluorescence signal increase. Figure 4*a,b* demonstrates bright field and fluorescence images of the GUV encapsulated biosensor at *t* = 0 and after approximately 4 h at 50 mM external lactate concentration. Microscopy images revealed non-encapsulated bacteria, which is a limitation of the system that can be improved upon in the future through microfluidic encapsulation strategies which offer greater encapsulation efficiencies [40,41], and through size-dependent filtration approaches. Nevertheless, the vesicles that encapsulated the microbes remained stable and the population of bacteria increased inside the vesicle over 4 h, as shown in figure 4*a*.

The response of the encapsulated biosensor under different lactate conditions was monitored in a bulk GUV sample using a fluorimeter. Bacteria in different concentrations of lactate grew to a similar number over time and tripled over 3 h in all conditions (electronic supplementary material, figure S3). In all experiments, a plateau in fluorescence was eventually reached, which was taken as the end-point. The higher the lactate concentration, the larger the end-point fluorescence produced by the biosensor (figure 4*d*), with maximum intensity being reached earlier for lower lactate concentrations (approx. 1 h for 0.05 mM, approximately 12 h for 15 mM). The control case, which was identical apart from the absence of lactate, showed no fluorescence increase over time, as expected.

To test the effect of non-encapsulated bacteria, we ran the same experiment with 15 mM lactate externally, but with no α-HL pores present (figure 4*e*). In this scenario, only the non-encapsulated bacteria would be exposed to lactate. Compared with when α-HL was present, the fluorescence intensity after 12 h was 54–60% lower, showing that, although non-encapsulated bacteria are contributing to the signal, the majority of the response is derived from bacteria that are encapsulated in the vesicle.

Based on these experiments, we extrapolated a calibration curve based on the response of the hybrid biosensor to distinct lactate concentrations (figure 4*c*), which showed an increase in fluorescence signal up to 15 mM with lactate concentration and a linear measurement range up to 5 mM. The response range is, therefore, larger than the enzymatic-based sensor (linear up to 0.2 mM) under the conditions used in our experiments.

## 4. Conclusion

In conclusion, we show that engineered bacteria can be used as functional modules within lipid vesicles. In our system, the chosen functionality was lactate biosensing, although this work paves the way for the wide array of capabilities of synthetic biology systems to be incorporated in future. Whole-cell biosensors have a number of advantages over other types of sensors, including increased sensitivity, reduced cost and robustness. The use of engineered organisms encapsulated within vesicle compartments has several potential benefits compared with their use in open conditions, arising from the presence of a lipid vesicle barrier. First, the vesicle can provide protection against surrounding environments in instances where these are non-compatible. Second, the chemical environments inside the vesicle can be optimized for the performance of the bio-module, without the need to change the composition of the bulk solution. Third, the vesicle provides a two-dimensional surface that can be easily engineered to give added functionality, e.g. through the addition of molecular recognition modules, the addition of light-, temperature- and pH-responsive modalities, and responsive pores and channels [7,42–45]. These features may be desirable in applications ranging from co-culture (where the encapsulated ‘sensor’ cells are shielded from the main culture solution) to targeted binding in medicinal settings (e.g. by functionalizing the vesicle lipid membrane with appropriate receptors).

## Supplementary Material

Supplementary Information

## Data accessibility

All relevant data are available from the authors on reasonable request.

## References

[1] Bally M, Bailey K, Sugihara K, Grieshaber D, Vörös J, Städler B 2010 Liposome and lipid bilayer arrays towards biosensing applications. Small 6, 2481–2497. (10.1002/smll.201000644) 20925039

[2] Daraee H, Etemadi A, Kouhi M, Alimirzalu S, Akbarzadeh A 2016 Application of liposomes in medicine and drug delivery. Artif. Cells Nanomed. Biotechnol. 44, 381–391. (10.3109/21691401.2014.953633) 25222036

[3] Koçer A 2007 A remote controlled valve in liposomes for triggered liposomal release. J. Liposome Res. 17, 219–225. (10.1080/08982100701528203) 18027242

[4] Mason JT, Xu L, Sheng Z-M, O'Leary TJ 2006 A liposome-PCR assay for the ultrasensitive detection of biological toxins. Nat. Biotechnol. 24, 555 (10.1038/nbt1201) 16617336

[5] Bolinger PY, Stamou D, Vogel H 2008 An integrated self-assembled nanofluidic system for controlled biological chemistries. Angew. Chem. Int. Ed. 47, 5544–5549. (10.1002/anie.200801606) 18613154

[6] Elani Y, Law RV, Ces O 2014 Vesicle-based artificial cells as chemical microreactors with spatially segregated reaction pathways. Nat. Commun. 5, 5304 (10.1038/ncomms6305) 25351716

[7] Hindley JW, Elani Y, McGilvery CM, Ali S, Bevan CL, Law RV, Ces O 2018 Light-triggered enzymatic reactions in nested vesicle reactors. Nat. Commun. 9, 1093 (10.1038/s41467-018-03491-7) 29545566 PMC5854585

[8] Luisi PL, Ferri F, Stano P 2006 Approaches to semi-synthetic minimal cells: a review. Naturwissenschaften 93, 1–13. (10.1007/s00114-005-0056-z) 16292523

[9] Salehi-Reyhani A, Ces O, Elani Y 2017 Artificial cell mimics as simplified models for the study of cell biology. Exp. Biol. Med. 242, 1309–1317. (10.1177/1535370217711441) PMC5528198 28580796

[10] Villar G, Graham AD, Bayley H 2013 A tissue-like printed material. Science 340, 48 (10.1126/science.1229495) 23559243 PMC3750497

[11] Walde P, Cosentino K, Engel H, Stano P 2010 Giant vesicles: preparations and applications. ChemBioChem. 11, 848–865. (10.1002/cbic.201000010) 20336703

[12] Parolini L, Mognetti BM, Kotar J, Eiser E, Cicuta P, Di Michele L 2015 Volume and porosity thermal regulation in lipid mesophases by coupling mobile ligands to soft membranes. Nat. Commun. 6, 5948 (10.1038/ncomms6948) 25565580 PMC4354032

[13] Manjappa AS, Chaudhari KR, Venkataraju MP, Dantuluri P, Nanda B, Sidda C, Sawant KK, Ramachandra Murthy RS 2011 Antibody derivatization and conjugation strategies: application in preparation of stealth immunoliposome to target chemotherapeutics to tumor. J. Control Release. 150, 2–22. (10.1016/j.jconrel.2010.11.002) 21095210

[14] Gopalakrishnan G, Danelon C, Izewska P, Prummer M, Bolinger PY, Geissbühler I, Demurtas D, Dubochet J, Vogel H 2006 Multifunctional lipid/quantum dot hybrid nanocontainers for controlled targeting of live cells. Angew. Chem. Int. Ed. 45, 5478–5483. (10.1002/anie.200600545) 16847983

[15] Bolognesi G, Friddin MS, Salehi-Reyhani A, Barlow NE, Brooks NJ, Ces O, Elani Y 2018 Sculpting and fusing biomimetic vesicle networks using optical tweezers. Nat. Commun. 9, 1882. 29760422 10.1038/s41467-018-04282-w PMC5951844

[16] Koçer A, Walko M, Meijberg W, Feringa BL 2005 A light-actuated nanovalve derived from a channel protein. Science 309, 755–758. (10.1126/science.1114760) 16051792

[17] Miller D, Booth PJ, Seddon JM, Templer RH, Law RV, Woscholski R, Ces O, Barter LM 2013 Protocell design through modular compartmentalization. J. R. Soc. Interface 10, 20130496 (10.1098/rsif.2013.0496) 23925982 PMC3758011

[18] Burns JR, Stulz E, Howorka S 2013 Self-assembled DNA nanopores that span lipid bilayers. Nano Lett. 13, 2351–2356. (10.1021/nl304147f) 23611515

[19] Elani Y, Law RV, Ces O 2015 Protein synthesis in artificial cells: using compartmentalisation for spatial organisation in vesicle bioreactors. Phys. Chem. Chem. Phys. 17, 15534–7. (10.1039/C4CP05933F) 25932977

[20] Noireaux V, Libchaber A 2004 A vesicle bioreactor as a step toward an artificial cell assembly. Proc. Natl Acad. Sci. USA 101, 17 669–17 674. (10.1073/pnas.0408236101) PMC539773 15591347

[21] Oberholzer T, Albrizio M, Luisi PL 1995 Polymerase chain reaction in liposomes. Chem. Biol. 2, 677–682. (10.1016/1074-5521(95)90031-4) 9383474

[22] Merkle D, Kahya N, Schwille P 2008 Reconstitution and anchoring of cytoskeleton inside giant unilamellar vesicles. ChemBioChem. 9, 2673–2681. (10.1002/cbic.200800340) 18830993

[23] Trantidou T, Friddin M, Elani Y, Brooks NJ, Law RV, Seddon JM, Ces O 2017 Engineering compartmentalized biomimetic micro- and nanocontainers. ACS Nano 11, 6549–6565. (10.1021/acsnano.7b03245) 28658575

[24] Lentini R et al. 2014 Integrating artificial with natural cells to translate chemical messages that direct *E. coli* behaviour. Nat. Commun. 5, 4012 (10.1038/ncomms5012) 24874202 PMC4050265

[25] Elani Y, Trantidou T, Wylie D, Dekker L, Polizzi K, Law RV, Ces O 2018 Constructing vesicle-based artificial cells with embedded living cells as organelle-like modules. Sci. Rep. 8, 4564 (10.1038/s41598-018-22263-3) 29540757 PMC5852042

[26] Tan Y-C, Hettiarachchi K, Siu M, Pan Y-R, Lee AP 2006 Controlled microfluidic encapsulation of cells, proteins, and microbeads in lipid vesicles. J. Am. Chem. Soc. 128, 5656–5658. (10.1021/ja056641h) 16637631

[27] Pautot S, Frisken BJ, Weitz D 2003 Production of unilamellar vesicles using an inverted emulsion. Langmuir 19, 2870–2879. (10.1021/la026100v)

[28] Wei TY, Cheng CM 2016 Synthetic biology-based point-of-care diagnostics for infectious disease. Cell Chem Biol. 23, 1056–1066. (10.1016/j.chembiol.2016.07.016) 27662252

[29] Karig DK 2017 Cell-free synthetic biology for environmental sensing and remediation. Curr. Opin. Biotechnol. 45, 69–75. (10.1016/j.copbio.2017.01.010) 28226291

[30] Dekker L, Polizzi KM 2017 Sense and sensitivity in bioprocessing-detecting cellular metabolites with biosensors. Curr. Opin. Chem. Biol. 40, 31–36. (10.1016/j.cbpa.2017.05.014) 28609710

[31] Rakszewska A, Tel J, Chokkalingam V, Huck WT 2014 One drop at a time: toward droplet microfluidics as a versatile tool for single-cell analysis. NPG Asia Mater. 6, e133 (10.1038/am.2014.86)

[32] Weaver JC, McGrath P, Adams S 1997 Gel microdrop technology for rapid isolation of rare and high producer cells. Nat. Med. 3, 583–585. 9142132 10.1038/nm0597-583

[33] Lim F, Sun AM 1980 Microencapsulated islets as bioartificial endocrine pancreas. Science 210, 908–910. (10.1126/science.6776628) 6776628

[34] Rabanel JM, Banquy X, Zouaoui H, Mokhtar M, Hildgen P 2009 Progress technology in microencapsulation methods for cell therapy. Biotechnol. Prog. 25, 946–963. (10.1002/btpr.226) 19551901

[35] Goers L, Ainsworth C, Goey CH, Kontoravdi C, Freemont PS, Polizzi KM 2017 Whole-cell *Escherichia coli* lactate biosensor for monitoring mammalian cell cultures during biopharmaceutical production. Biotechnol. Bioeng. 114, 1290–1300. (10.1002/bit.26254) 28112405 PMC5412874

[36] Fujii S, Matsuura T, Sunami T, Nishikawa T, Kazuta Y, Yomo T 2014 Liposome display for in vitro selection and evolution of membrane proteins. Nat. Protoc. 9, 1578–1591. (10.1038/nprot.2014.107) 24901741

[37] Trantidou T, Elani Y, Parsons E, Ces O 2017 Hydrophilic surface modification of PDMS for droplet microfluidics using a simple, quick, and robust method via PVA deposition. Microsyst. Nanoeng. 3, 16091 (10.1038/micronano.2016.91) PMC6444978 31057854

[38] Song L, Hobaugh MR, Shustak C, Cheley S, Bayley H, Gouaux JE 1996 Structure of staphylococcal alpha-hemolysin, a heptameric transmembrane pore. Science 274, 1859–1865. (10.1126/science.274.5294.1859) 8943190

[39] Zhou M, Diwu Z, Panchuk-Voloshina N, Haugland RP 1977 A stable nonfluorescent derivative of resorufin for the fluorometric determination of trace hydrogen peroxide: applications in detecting the activity of phagocyte NADPH oxidase and other oxidases. Anal. Biochem. 253, 162–168. (10.1006/abio.1997.2391) 9367498

[40] van Swaay D 2013 Microfluidic methods for forming liposomes. Lab. Chip. 13, 752–767. (10.1039/c2lc41121k) 23291662

[41] Elani Y 2016 Construction of membrane-bound artificial cells using microfluidics: a new frontier in bottom-up synthetic biology. Biochem. Soc. Trans. 44, 723–730. (10.1042/BST20160052) 27284034 PMC4900754

[42] Mura S, Nicolas J, Couvreur P 2013 Stimuli-responsive nanocarriers for drug delivery. Nat. Mater. 12, 991 (10.1038/nmat3776) 24150417

[43] Karamdad K, Hindley JW, Bolognesi G, Friddin MS, Law RV, Brooks NJ, Ces O, Elani Y 2018 Engineering thermoresponsive phase separated vesicles formed via emulsion phase transfer as a content-release platform. Chem. Sci. 9, 4851–4858. (10.1039/C7SC04309K) 29910937 PMC5982195

[44] Al-Ahmady ZS, Al-Jamal WT, Bossche JV, Bui TT, Drake AF, Mason AJ, Kostarelos K 2012 Lipid–peptide vesicle nanoscale hybrids for triggered drug release by mild hyperthermia in vitro and in vivo. ACS Nano 6, 9335–9346. (10.1021/nn302148p) 22857653 PMC3480335

[45] Leung SJ, Romanowski M 2012 Light-activated content release from liposomes. Theranostics 2, 1020 (10.7150/thno.4847) 23139729 PMC3493200

