# Supplementary material for: Functionalizing cell-mimetic giant vesicles with encapsulated bacterial biosensors
Source: Interface Focus. 2018 Aug 17;8(5):20180024. doi: 10.1098/rsfs.2018.0024 (PMC6227772; doi:10.1098/rsfs.2018.0024)
Supplement: Supplementary Information [file rsfs20180024supp1.docx]

**Supplementary Information**

**Functionalizing cell mimetic giant vesicles with encapsulated bacterial biosensors**

**Tatiana Trantidou^1^**^†^**, Linda Dekker^2^**^†^**, Karen Polizzi^2^, Oscar Ces^1,3,4^ * & Yuval Elani ^1,3,4^ ***

*^1^Department of Chemistry, Imperial College London, London, SW7 2AZ, UK*

*^2^Department of Life Sciences and Centre for Synthetic Biology and Innovation, Imperial College London, London, SW7 2AZ, UK*

*^3^Institute of Chemical Biology, Imperial College London, London, SW7 2AZ, UK*

*^4^ fabriCELL, Imperial College London, London, SW7 2AZ, UK*

^†^ These authors contributed equally to this work.

* Corresponding authors: Yuval Elani ([yuval.elani10@imperial.ac.uk](mailto:yuval.elani10@imperial.ac.uk)) and Oscar Ces ([o.ces@imperial.ac.uk](mailto:o.ces@imperial.ac.uk)).

| **Table S1.** Sequence of IIdPRD promoter. | |  |
| --- | --- | --- |
| Promoter | Sequence (5’ → 3’) | |
| Natural promoter sequence | CTTTACCAGACATCTCCCCCCACAAGAATTGGCCCTACCAATTCTTCGCTTATCTGACCTCTGGTTCACAATTTCCCAATTAAAACTC  ACATCAATGTTGCCAATACATAACATTTAGTTAACCATTCATTGTCATTATCCCTACACAACACAATTGGCAGTGCCACTTTTACACAA  CGTGTGACAAGGAGATGAGCAACAGACTCATTACACGATGTGCGTGGACTCC | |
| Synthetic promoter sequence | CTTTACCAGACATCTCCCCCCACAAGAATTGGCCCTACCAATTCTTCGCTTATCTGACCTCTGGTTCACAATTTCCCAATTAAAACTC  ACATCAATGTTGACAGCTAGCTCAGTCCTAGGGATTGTGCTAGCTCATTATCCCTACACAACACAATTGGCAGTGCCACTTTTACAC  AACGTGTGACAAGGAGATGAGCAACAGACTCATTACACGATGTGCGTGGACTCC | |
|  | |  |
|  | |  |


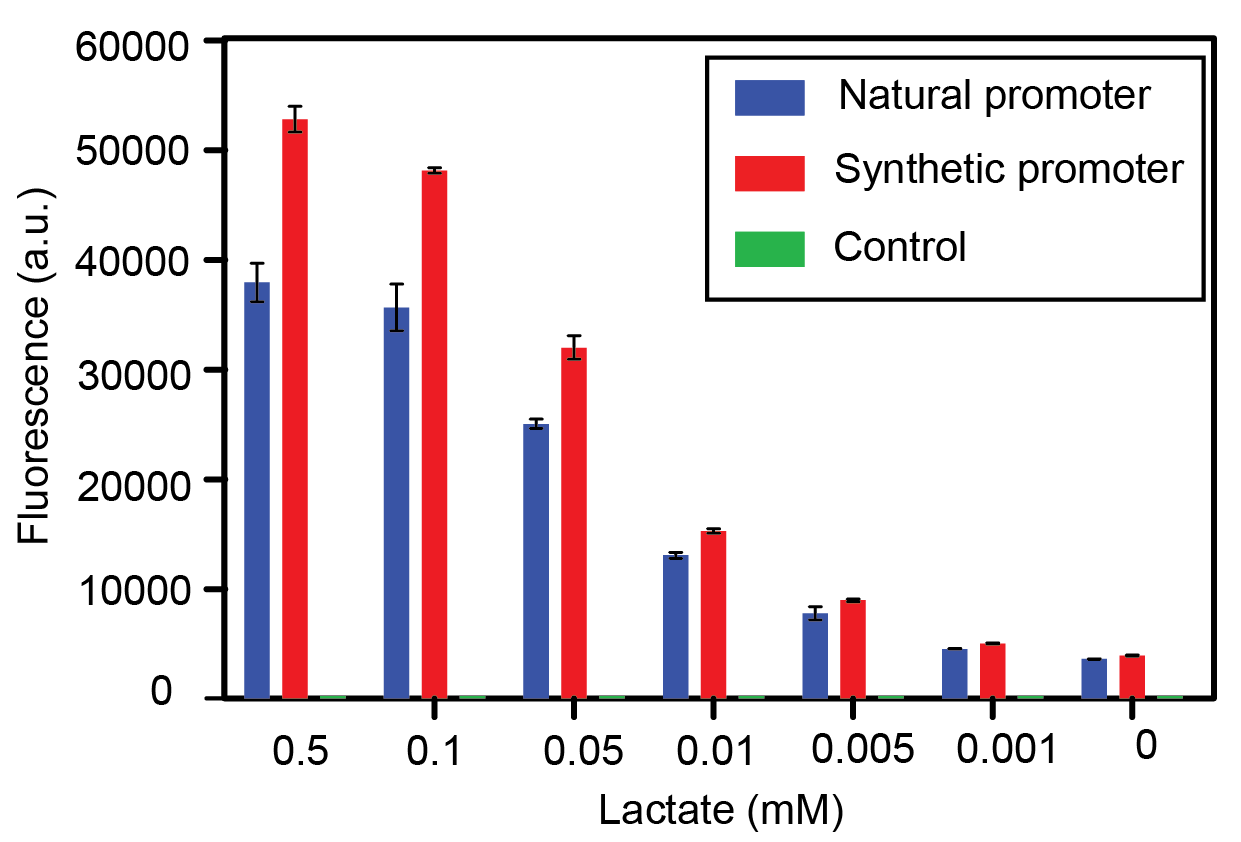
**Figure S1** Comparison between the lactate biosensor containing a synthetic lldPRD promoter (red) with the lactate biosensor containing the natural lldPRD promoter (blue). The whole-cell *E. coli* biosensor and control cells were spiked with different concentrations of L-lactate. Fluorescence intensity was measured using a flow cytometer.

**
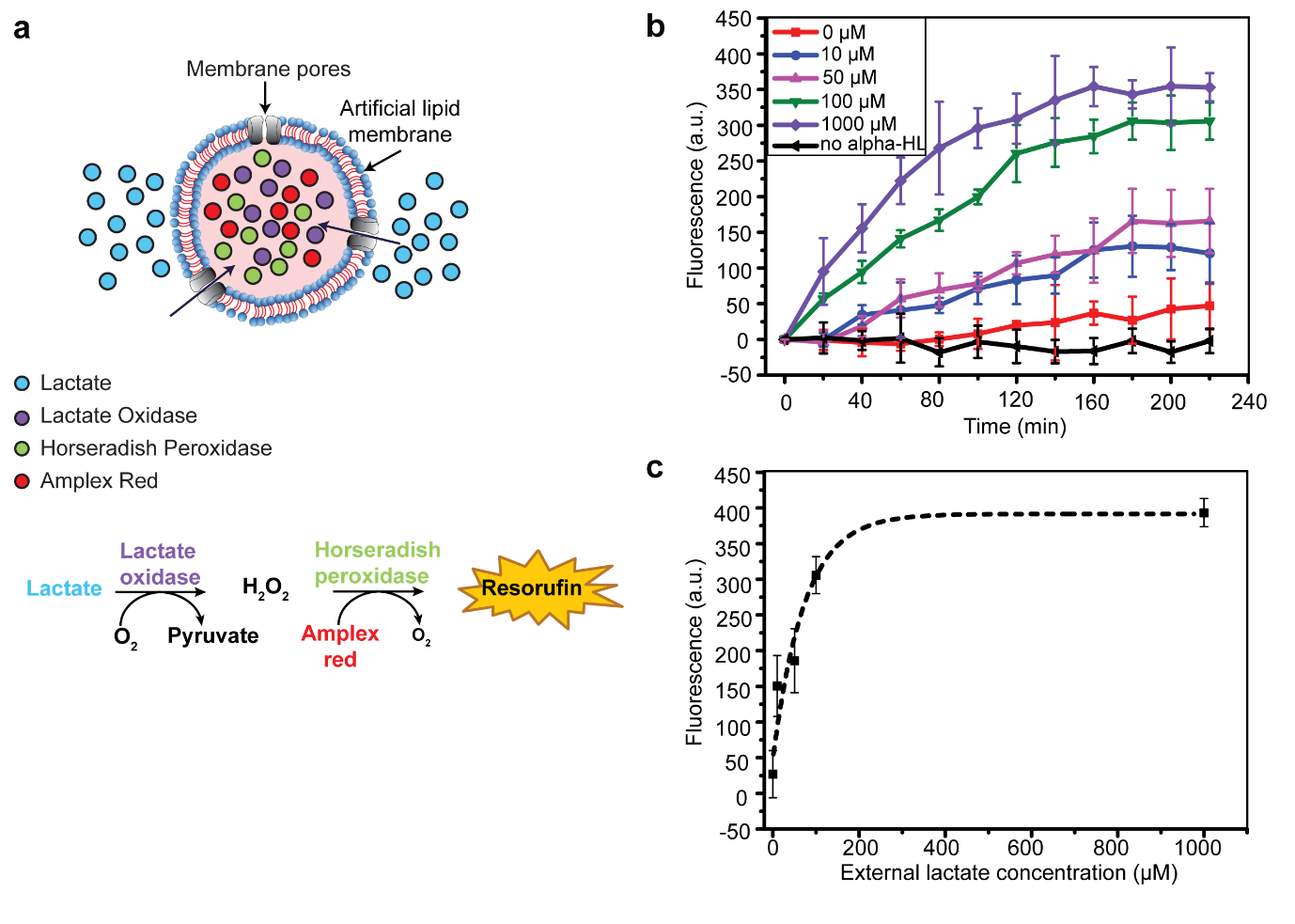
**

**Figure S2** (a) Schematic of the vesicle system that was generated to assess lactate permeation via the alpha-HL pores, and multi-step enzymatic scheme. Lactate enters the vesicle, where it is oxidised, producing hydrogen peroxide, which translocates through the bilayer to initiate the oxidation of Amplex Red. (b) Graph showing fluorescence intensity of the Amplex Red dye being oxidised to fluorescent resorufin after the full two-step reaction cascade takes place. (c) Characterisation data at different concentrations of lactate. Error bars represent the standard deviation of 5 technical replicates.


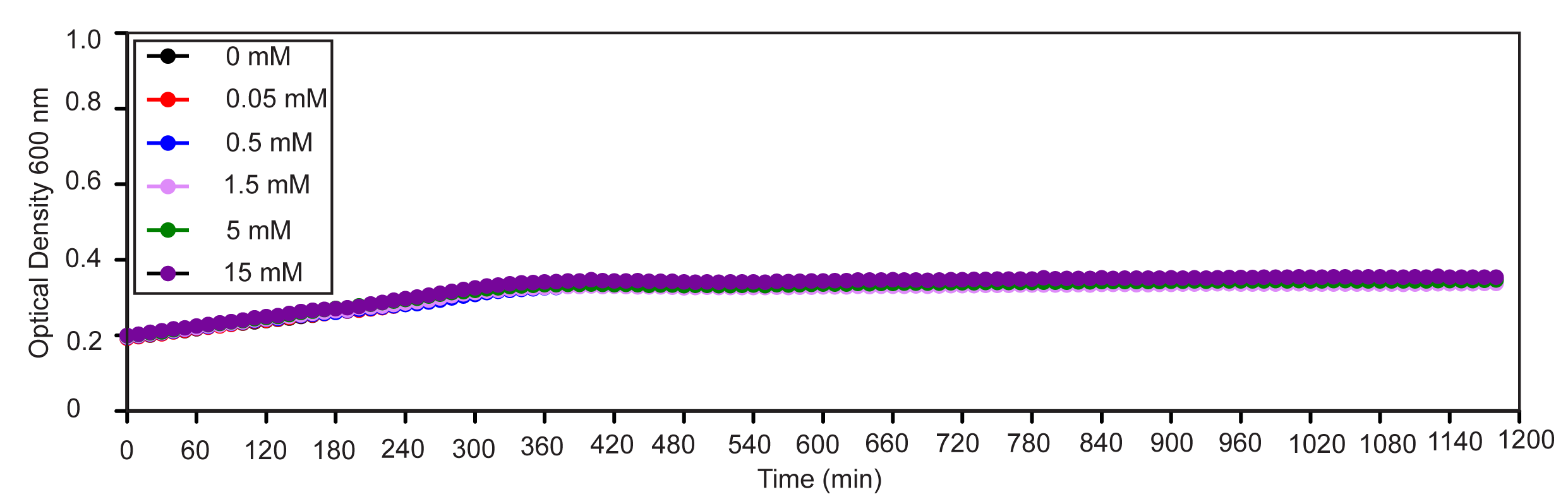


**Figure S3** Optical density (600 nm) measured from encapsulated *E. coli* in GUVs, indicating that cells grew to a similar number over time in different lactate concentrations.
